# Motor-Enriched Encoding Can Improve Children’s Early Letter Recognition

**DOI:** 10.3389/fpsyg.2020.01207

**Published:** 2020-06-26

**Authors:** Linn Damsgaard, Sofie Rejkjær Elleby, Anne Kær Gejl, Anne Sofie Bøgh Malling, Anna Bugge, Jesper Lundbye-Jensen, Mads Poulsen, Glen Nielsen, Jacob Wienecke

**Affiliations:** ^1^Department of Nutrition, Exercise and Sports, University of Copenhagen, Copenhagen, Denmark; ^2^Department of Midwifery, Physiotherapy, Occupational Therapy and Psychomotor Therapy, University College Copenhagen, Copenhagen, Denmark; ^3^Department of Nordic Studies and Linguistics, University of Copenhagen, Copenhagen, Denmark

**Keywords:** motor-enriched, academic learning, children, cognition, letter recognition

## Abstract

It is not known how effective specific types of motor-enriched activities are at improving academic learning and early reading skills in children. The aim of this study was to investigate whether fine or gross motor enrichment during a single session of recognizing letters “b”/“d” can improve within-session performance or delayed retention the following day in comparison to letter recognition practice without movement. Furthermore, the aim was to investigate children’s motivation to perform the specific tasks. We used a randomized controlled intervention study-design to investigate the effect of 10-min motor-enriched “b”/“d” letter training on children’s ability to recognize the letters “b” and “d” (*n* = 127, mean age = 7.61 ± SD = 0.44 years) acutely, and in a delayed retention test. Three groups were included: a fine motor-enriched group (FME), a gross motor-enriched group (GME), that received 10 min of “b” and “d” training with enriched gestures (fine or gross motor movements, respectively), and a control group (CON), which received non motor-enriched “b”/“d” training. The children’s ability to recognize “b” and “d” were tested before (T0), immediately after (T1), and one day after the intervention (T2) using a “b”/“d” Recognition Test. Based on a generalized linear mixed model a significant group-time interaction was found for accuracy in the “b”/“d” Recognition Test. Specifically, FME improved their ability to recognize “b”/“d” at post intervention (T0→T1, *p* = 0.008) and one-day retention test (T0→T2, *p* < 0.001) more than CON. There was no significant difference in change between GME and CON. For reaction time there were no significant global interaction effects observed. However, planned post hoc comparisons revealed a significant difference between GME and CON immediately after the intervention (T0→T1, *p* = 0.03). The children’s motivation-score was higher for FME and GME compared to CON (FME-CON: *p* = 0.01; GME-CON: *p* = 0.01). The study demonstrated that fine motor-enriched training improved children’s letter recognition more than non motor activities. Both types of motor training were accompanied by higher intrinsic motivation for the children compared to the non motor training group. The study suggests a new method for motor-enriched letter learning and future research should investigate the underlying mechanisms.

## Introduction

The acquisition and development of reading skills is a central cognitive attribute to society. How much a student reads is a unique and powerful contributor to a variety of academic skills, including oral language, basic reading skills, spelling, content/declarative knowledge, and vocabulary skills (Sparks et al., 2014). Children who learn to recognize and name the letters early demonstrate stronger decoding skills and reading comprehension in later grades (Hammill, 2004; Schatschneider et al., 2004; Piasta et al., 2012). Thus, it is important to identify strategies for improving early reading skills in children.

Development of single word reading can be described as moving through a series of overlapping phases. Here, children successively learn more advanced means for recognizing and learning orthographic words (Ehri, 2008). In the pre-alphabetic phase, children recognize words by their general shape and contextual cues. This strategy has the important limitation that it does not allow the child to read unknown words. In the partial alphabetic phase, word recognition is supported by knowledge of some letters and some letter sounds. In the full alphabetic phase, the child knows all the letters and their sounds and how to blend the sounds into full word pronunciations. This allows them to read unfamiliar words. In the consolidation phase, this process is extended to chunks of letters, for example, representing specific syllables and morphemes. Thus, in all phases past the pre-alphabetic phase, recognition and differentiation of individual letters plays an important role in recognizing whole words (Ehri, 2008).

The ability to recognize letters is therefore important to early literacy development (Hiebert et al., 1984). Especially children’s ability to recognize letter names helps them to learn sounds, which requires letter knowledge (Hammill, 2004; Foulin, 2005; Bara and Bonneton-Botté, 2018). Letter naming knowledge is a predictor of learning how to read based upon longitudinal correlations between letter naming and reading achievement in children (Kirby et al., 2008). Weak letter sound knowledge is known to cause difficulties in translations from reading to speaking, and is an important component to pay attention to in order to help children learn to read (Hulme et al., 2012). A study by Badian (2005) concluded that children (8–10 years) with a deficit in recognizing errors in letter orientation were poorer readers than children without this deficit (Badian, 2005). It has been shown that children often struggle especially with learning the letters “b” and “d” and often make a lot of orientation errors with these two letters (Tortorelli et al., 2017). Thus, it is relevant to design a short-term intervention focusing on improving children’s ability to recognize and discriminate “b” and “d” (Badian, 2005).

Research shows that motivation plays a central role in literacy development. Motivation can be facilitated by positive learning environments and positive reading experiences (Gambrell, 1996; Chapman and Tunmer, 1997; Guthrie et al., 1999; Marinak et al., 2015). There are different types of motivation with different levels of autonomy and different effects on academic achievement and development (Gottfried, 1985; Ryan and Deci, 2000). Intrinsic motivation implies doing something because it is enjoyable and interesting, more than doing something based upon pressure. Feelings of competence, autonomy, and relatedness are basic psychological needs, which must be fulfilled for sustaining the intrinsic motivation (Deci et al., 1999). It is known that especially intrinsic motivation is associated with higher reading achievements, higher conceptual understanding, and that this type of motivation generates higher ability to persevere when reading tasks become challenging (Gottfried, 1985; Marinak et al., 2015). Classroom based physical activities have shown to be a strategy to increase intrinsic motivation, moreover, fostering executive functions (Vazou and Smiley-Oyen, 2014), especially when the activities are built upon deliberate play (Pesce et al., 2016). Accordingly, several interventions have focused on the potential positive impact of physical activity breaks in addition to cardiovascular exercise to facilitate cognitive performance (for reviews, see Hillman et al., 2008; Pesce and Ben-Soussan, 2015; Pesce et al., 2016), and behavioral effects (Voelcker-Rehage and Niemann, 2013).

Intervention studies with focus on quantitative characteristics of physical activity with the purpose of improving cognitive performance and academic achievement have received a lot of attention (Best, 2010; Donnelly et al., 2016). Less focus has been on more qualitative characteristics of physical activity, where coordinative activities are used within learning sessions (Diamond, 2015). Motor-enriched encoding is one type of learning-model, which could be used.

Motor-enriched encoding, where learning of a subject is combined with meaningful motor activities, has previously shown positive effects in a wide range of different learning paradigms (Beck et al., 2016), for example, conceptual and associative learning, action-sentence learning, and also more academically related vocabulary and foreign language learning (Macedonia and Klimesch, 2014; Mavilidi et al., 2015; Mayer et al., 2015). In both children and adults motor-enriched foreign word learning is facilitated when congruent gestures are used (Macedonia et al., 2011; Mavilidi et al., 2015; Toumpaniari et al., 2015). Mavilidi et al. (2015) demonstrated that pre-school children (age of 5 years old), before the full alphabetic phase, also benefitted from motor-enriched foreign word learning, however, whether letter learning is also positively influenced by pairing encoding with congruent gestures before the full alphabetic phase is not known. Potential positive learning effects of motor-enriched encoding might have several explanations also involving working memory and retrieval processes. One theory is that motor-enriched activities activate not only cortical areas of cognitive control but also a wide range of cortical motor and sensory areas (Engelkamp and Zimmer, 1989; Barsalou, 2008; Macedonia, 2019). These cortical areas are responsible for generation of actions and processing the sensory consequences of the actions this may help encoding by (a) efficient activation of both the visual and phonological subsystems supporting working memory and (b) increased activation of sensorimotor brain areas both during encoding and retrieval (Macedonia et al., 2011; Mayer et al., 2015; Macedonia, 2019). The latter has been shown by Mayer et al. (2015) using functional magnetic resonance imaging (fMRI), reporting that a word recall task at 2-months post-acquisition correlates with increased activation of the left-brain motor cortex and the temporal brain sulcus in adults. The findings by Mayer et al. (2015) supported the multisensory learning theory proposed by Shams and Seitz (2008) that words encoded audio-visually improve performance compared to words encoded audibly (Shams and Seitz, 2008; Mayer et al., 2015). It is assumed that dividing the cognitive load imposed by a learning task across different working memory subsystems (visual and auditory) can prevent negative effects of too high load on one specific subsystem (Baddeley, 2010).

A study within mathematics has shown that hand gesturing during problem solving can prevent too high load on one specific subsystem (Goldin-Meadow et al., 2001). Goldin-Meadow et al. (2001) investigated how gesturing during one task (children had to explain solution to a math problem) impacted performance on another task (remembering words), and showed that children (mean age = 9.91 years) remembered more items when gesturing compared to non-gesturing (Goldin-Meadow et al., 2001).

Interestingly, Mavilidi et al. (2015) found that the effect is dependent on the motor modality used. The study compared whole body motor activity and part body activity (arms and hands) integrated into the academic content. The whole-body activities resulted in the highest scores in a free-recall and cued-recall vocabulary task in preschool children. These findings indicate that there may be a difference in efficacy between the use of fine motor movements and gross motor movements during learning. A recent review identifies a knowledge gap in terms of when and how to incorporate gross motor movement in academic lessons (Mavilidi et al., 2018). The authors state that when movements are not meaningfully or congruently incorporated in the academic lesson, children have more difficulty performing a word recall task (Mavilidi et al., 2015; Toumpaniari et al., 2015). Research on motor-enriched encoding has predominantly focused on word learning and solving math problems while few studies have focused on letter recognition and if so they predominantly focus on writing and tracing letters (Hulme et al., 1987; Bara and Bonneton-Botté, 2018) or have mixed encoding strategies (Kirk and Kirk, 2016). It is, however, not known if motor-enriched encoding can improve reading abilities or letter recognition. The present study will further elucidate this topic of meaningfully integrating fine and gross motor movements into the learning activities focusing on the distinction between “b” and “d.”

Based upon current knowledge regarding the benefit of motor-enriched encoding, we hypothesize that 10 min of fine or gross motor-enriched activities can improve recognition of “b” and “d” and distinction between these letters and furthermore higher intrinsic learning motivation for the learning activity more than non motor control activities.

## Materials and Methods

### Participants

**FIGURE 1:**
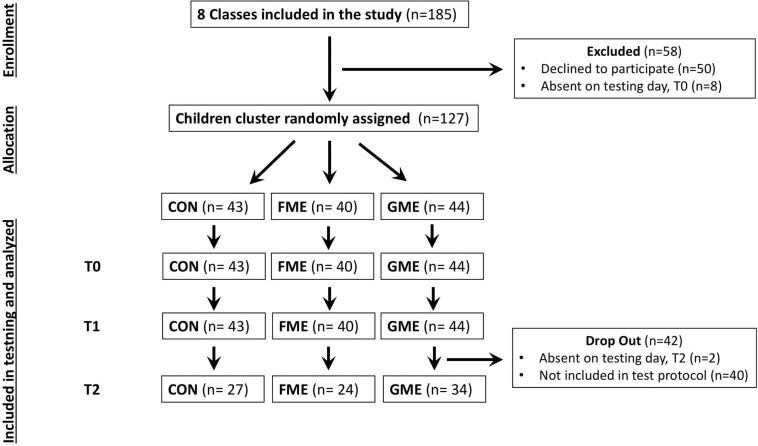
Flow diagram of the study. Retention test (T2) was not performed due to practical issues at one school, which means that 40 children were not performing T2 “b”/“d” Recognition Test.

The study was conducted with first grade children recruited from eight different classes from three elementary schools in the Copenhagen area. In total 127 children (71 girls and 56 boys, mean age ± SD = 7.61 ± 0.44 years) were included in the study after obtaining written consent from parents, corresponding to 73% of the invited children (see Table 1 for demographic characteristics within each group). Retention test (T2) at one of the schools was not performed due to practical issues which means that 40 children were excluded from the “b”/“d” Recognition Test at T2. In addition, two children were absent at the day of T2 (Figure 1). The study was approved by the local Ethical Committee at University of Copenhagen, Denmark (protocol: 504-0032/18-5000), and was carried out in accordance with the Helsinki Declaration II.

**TABLE 1:** Demographics for the three intervention groups (CON, FME, GME).

	CON	FME	GME
Participants (*n*)	43	40	44
Age (Years)	7.61 ± 0.44	7.61 ± 0.44	7.62 ± 0.44
Sex (% Boys)	46.51	40.00	45.45
Bilingualism (% Bilingual)	25.58	27.50	27.27
Dominant hand (R, %)	90.70	92.68	88.64

*Data reported as mean ± SD. No significant between-group differences were observed for any of the measures. CON, control group; FME, fine motor-enriched group; GME, gross motor-enriched group.*

### Intervention

All participating children were randomized individually before baseline assessment to receive one of three “b”/“d” training sessions: a fine motor-enriched (FME), a gross motor-enriched (GME) or non motor (CON) condition. All three groups received one-to-one teaching focusing on “b”/“d” for 10 min with one investigator. Budde et al. (2008) demonstrated that 10 min coordinative exercise resulted in a higher attention score than normal sport activities. Based on Budde and colleagues’ results the intervention duration of 10 min was chosen for this present study.

**FIGURE 2:**
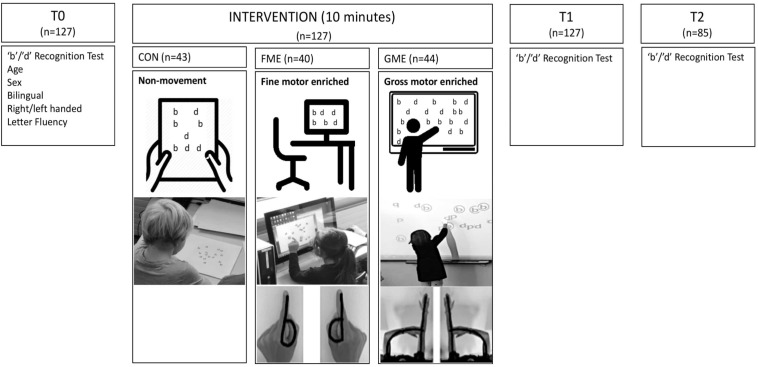
Intervention overview. At T0 a “b”/“d” Recognition Test were performed and participants demographics were collected. All participating children were randomized into three groups: a fine motor-enriched group (FME) and a gross motor-enriched group (GME), that received “b” and “d” activities enriched with gestures as fine and gross motor movements, respectively, and a control group (CON), which received non motor-enriched “b”/“d” teaching. Pictures shows how the children were instructed to use their hands and arms in the FME and GME group. At T1 children performed a “b”/“d” Recognition Test. The next day (after 20–24 h) another “b”/“d” Recognition Test was completed (T2).

The training was not blinded to neither participants nor the investigators. The groups differed in the teaching approach. Children in the FME group were sitting at a table with their elbows resting at the table while trying to find the letters “b” and “d” randomly distributed on a computer screen, while simultaneously pronouncing the name of the letter they touched with their fingers. Using their fingers, the children shaped the letters “b” with left hand fingers and “d” with right hand fingers before touching the screen (Figure 2). The GME group was standing at a smartboard and created “b” with their left arm stretched out in the front of the body and their right hand placed at the left elbow. They created “d” by stretching out their right arm in front of the body and placing their left hand at the right elbow (Figure 2). Further, they were asked to touch the “b” or “d” on the smartboard, while saying the name of the letter they touched. The CON group searched for the letter’s “b” and “d” on a white A4 paper while remaining silent. They were instructed to sit with their arms resting on the table through the 10-min session and were restricted only to use their eyes during the intervention. The investigator carefully observed the children’s tracking of “b”/“d”s and helped change the paper, so the children did not have to move (Figure 2). All children thoroughly performed the task and no oral feedback was given to any of the three groups. The exact duration and the number of trials completed during intervention were registered.

### Test Procedures

Age, sex, handedness, and bilingualism of the children are known to influence reading abilities (Lundberg et al., 2012) and were collected prior to baseline measures. For all three groups (CON, FME, and GME), baseline measures (T0) consisted of a letter fluency test and “b”/“d” Recognition Test. The children then completed one of the three interventions (CON, FME, and GME) and were post-evaluated in “b”/“d” Recognition Test and motivation (T1). The next day (after 20–24 h) another “b”/“d” Recognition Test was completed (T2). The intervention was conducted by six trained investigators. Each investigator performed “b”/“d” training for participants in all three groups (CON, FME, and GME) and performed all the measurements and intervention with the same child.

## Measures

### Letter Fluency

To estimate the children’s letter recognition at T0 a Danish version of letter fluency test was used (Good and Kaminski, 2002; Poulsen and Jensen, 2015). The test consisted of 116 uppercase and lowercase letters placed in rows of 10 letters on white A4 paper. The children were told to name as many letters as possible in 1 min. The test was conducted individually under supervision from the investigator. To get familiar with the test, a pre-test consisting of 10 letters were administered to the child before the actual test. The total number of all correctly named letters from the main test were used as an outcome measure.

### “b”/“d” Recognition Test

A simple “b”/“d” Recognition Test was developed for the present study by the research group. The “b”/“d” Recognition Test was applied to evaluate the children’s ability to recognize the letters “b” and “d.” A Cronbach’s Alpha coefficient was used to test the reliability of the “b”/“d” Recognition Test from T0, T1 and T2 (α = 0.7).

The test was completed on a computer in a one-to-one session between the child and an investigator. The children were comfortably placed in front of a 13.3-inch laptop at a distance that allowed them to press the response buttons on the keyboard with their index fingers, with their elbows resting on the edge of the table.

The laptop presented the stimuli using E-prime (Psychology Software Tools, Pittsburgh, PA, United States). Stimuli “b”/“d” letters were black 90 mm × 10 mm letters. The stimuli letters were d, b, p, q, m, and n, and presented in the center of the screen on a white background. The children completed a single block of 32 letters at T0/T1 (10 b, 10 d, and 12 p/q/m/n) and 60 letters for timepoint T2 (20 b, 20 d, and 20 p/q/m/n) with a stimulus duration of 3000 ms in random order. Prior to the actual test, a familiarization trial of six letters (b, d, p, q, m, and n) was completed, ensuring task compliance. The children were instructed to respond as precise and quickly as possible by pressing the left ctrl-key on keyboard when a “b” appeared on the screen, and the right-arrow-key on the keyboard when a “d” occurred at the laptop screen. Before each letter, “#” appeared in 700 ms as a ready signal. The children’s response latency and accuracy were logged. Total numbers correctly identified “b” and “d” and mean reaction time for correct trials were used as an estimate of the children’s learning effect of the intervention. All results more than ±2SD from the mean were considered outliers due to misunderstanding and excluded from the analyses (total excluded = 17 trials).

### Motivation

To measure the children’s intrinsic motivation for the “b” and “d” learning activities the Interest/Enjoyment scale of the Post-Experimental Intrinsic Motivation Inventory (IMI; McAuley et al., 1989) was used in the immediate post evaluation (T1). This questionnaire measures participants’ subjective experience related to an activity in an experiment. The Interest/Enjoyment subscale measures participants’ interest and enjoyment while performing a given activity and has been found to be a valid self-reported measure of intrinsic motivation (McAuley et al., 1989; Markland and Hardy, 1997). This scale has previously been used in other studies on the motivational effect of integrating physical and learning activities in primary schools (Vazou et al., 2012).

For the present study, the original seven items in the IMI Interest/enjoyment scale were translated into Danish using a translation-backtranslation process (Streiner and Norman, 2008). Because the scale was used in children the original 7-point response-scale was converted to a 4-point scale (1, not true at all; 2, only slightly true; 3, almost true; 4, true).

The investigator read the questions aloud for the children one by one. A mean (see Table 2) from the seven questions was calculated for each child and used as a measure for the Intrinsic motivation score.

**TABLE 2:** Performance at T0, T1, and T2 for the three intervention groups.

Measures	CON			FME			GME		
			
	T0	T1	T2	T0	T1	T2	T0	T1	T2
**General Test Information**									
Intervention Length (min)	9.44 ± 0.65			9.40 ± 0.69			9.75 ± 0.85		
Number of Trials	24.61 ± 13.23^§^			11.10 ± 3.92^§^			5.02 ± 1.76		
Letter Fluency	66.06 ± 17.19^###^			57.0 ± 19.28			55.06 ± 18.96		
**Letter recognition (*n* = 127)**									
“b”/“d” Score (% Correct Answers)	69.6 ± 43	85.8 ± 25*	89.2 ± 16**^,^***	73.7 ± 39	93.9 ± 12*^,#^	96.5 ± 6**^,^***^,##^	67.6 ± 45	88.8 ± 2*	89.2 ± 18**^,^***
**Reaction time**									
Estimated Marginal Means (ms)	948 ± 214.8	875 ± 335.7	823 ± 208.0**	854 ± 214.77	787 ± 237.9*	694 ± 154.5**	850 ± 226.49	780 ± 218.2*^,#^	704 ± 169.4**
**Motivation**									
IMQ-Score	3.36 ± 0.51			3.67 ± 0.30^§§^			3.64 ± 0.35^§§^		

Data reported as mean ± SD. “b”/“d” score (% correct answers) are calculated from the statistical model. CON, control group; FME, fine motor-enriched group; GME, gross motor-enriched group. *Indicates a significant difference within group from T0 to T1. **Indicates a significant difference within group from T0 to T2. ***Indicates a significant difference within group from T1 to T2. ^#^Indicates a significant difference in change from T0 to T1 between CON and GME/FME. ^##^Indicates a significant difference in change from T0 to T2 between CON and FME. ^###^Indicates a significant difference between CON and GME in Letter Fluency. ^§^ Indicates a significant difference between CON and GME and between FME and GME in amount of trials. ^§§^ Indicates a significant difference between FME and GME compared to CON in IMQ-Score.

### Statistical Analysis

The statistical analyses were performed in R Studio (R Core Team, Vienna, Austria).

Baseline characteristics were compared between groups using one-way analysis of variance and chi-square tests for categorical measures (bilingual, dominant hand, and sex). One-way ANOVA was used to identify possible group differences in baseline characteristics (Letter Fluency, age, “b”/“d” Recognition Score at T0, number of games and interventions length). If the one-way ANOVA revealed a significant difference (*p* < 0.05) a Tukey single-step adjusted multiple comparison of means was carried out to classify between which groups the differences were observed. Model validation was based upon visual inspection of residual plots and probability plots.

Data from the “b”/“d” Recognition Test were analyzed using generalized linear mixed model with group-time interactions as fixed effects, using R package lme4 (Bates et al., 2015). Since it was possible to score between 0 and 20 for correctly identified letters in the “b”/“d” Recognition Test at timepoint T0 and T1, and 0 and 40 in the T2 test, a general linear mixed model with a binomial distribution fitted the obtained correctly identified letters. The data was analyzed for group x time interactions with CON, FME, GME as groups and time were T0, T1, and T2. To account for the cluster structure and the repeated measures in the data “subjects” and “school” was added as random-effects and “age” as fixed effect, due to the testing period, since children’s letter recognition and letter-mirroring are age-dependent (Cornell, 1985).

Ratio Tests were used to reveal group x time interaction effects for accuracy and reaction time for correct “b”/“d” responses. Subsequently, if the test for interaction was significant, pairwise comparisons between delta values (on log odds scale) were used to characterize the interaction effect. To reduce the problem of multiple testing, only relevant model-based specified comparisons were performed including the comparisons of interest (time and group differences) using the *emmeans* R-package.^1^ *P*-value adjustment was based upon the Tukey method for comparing a family of three estimates.

Motivation scores were compared between groups by the non-parametric Kruskal-Wallis rank sum test. If the Kruskal–Wallis rank sum test revealed a significant difference (*p <* 0.05) pairwise Wilcoxon rank sum test was used post-hoc to investigate within-group differences. The test does not require the assumption of normal distribution. Pearson correlation coefficient was calculated to see correlation between accuracy in letter recognition and motivation score.

For all tests, a significance level of 0.05 was applied. Data are reported as means ± SD unless otherwise stated.

## Results

### Baseline Characteristics

The one-way-ANOVA revealed no significant between-group differences for the variables age (*p* = 0.63), “b”/“d” recognition (*p* = 0.66), bilingual (*p* = 0.97), dominant hand (R) (*p* = 0.83), or sex (*p* = 0.88) at T0. However, significant between-group differences at T0 was found in Letter Fluency and trial repetitions (Table 2). Specifically, CON performed significantly better compared to GME in Letter Fluency (*p* = 0.01) at baseline. Moreover, within the 10 min intervention CON performed more trials compared to GME (*p* = 0.04) and FME performed more trials compared to GME (*p* = 0.03). No correlation was found between the score in letter fluency and children’s ability to recognize “b”/“d” at T0, indicating that the score does not influence the main focus in this article. The same was observed with amount of trials and “b”/“d” recognition (*p* = 0.06) at T1 and T2. Therefore, the differences in the amount of trials may not have influenced the ability to recognize letters. Finally, no correlation was observed for correct recognized letters in the “b”/“d” task at all timepoint and time spend on the task (*p* > 0.05).

### Performance in Accuracy of “b”/“d” Recognition

**FIGURE 3:**
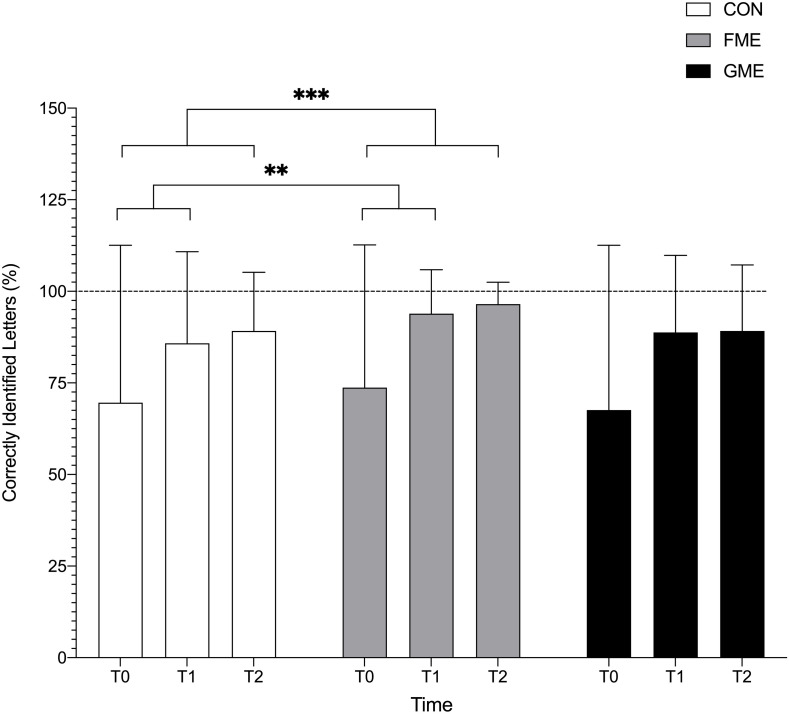
“b”/“d” letter recognition accuracy. Data reported as % correctly identified “b”/“d” letters. The percentage is made upon the statistical model. The line represents 100% correctly identified letters. All groups improved their total correct score (accuracy) to recognize and distinguish the letters “b” and “d” from T0 to T1 (*p* < 0.0001) and T0 to T2 (*p* < 0.0001) (not annotated in the illustration). The fine motor-enriched (FME) intervention had a greater improvement compared to CON from T0 to T1 (p = 0.008) and from T0 to T2 (*p* < 0.001). ^∗∗^, ^∗∗∗^ indicates significant improvement.

Likelihood Ratio Test showed a global significant interaction between time and groups (*p* < 0.0005) for accuracy of “b”/“d” recognition. A significant interaction was found for FME compared to CON from T0 to T1 (*p* = 0.008) and from T0 to T2 (*p* < 0.001). All groups significantly improved accuracy in “b”/“d” recognition from T0 to T1 and from T0 to T2 (*p* < 0.0001) (Figure 3). This implies that FME improved children’s performance in recognition “b”/“d” more compared to CON from T0 to T1 and T0 to T2.

### Performance in Reaction Time for “b”/“d” Recognition

**FIGURE 4:**
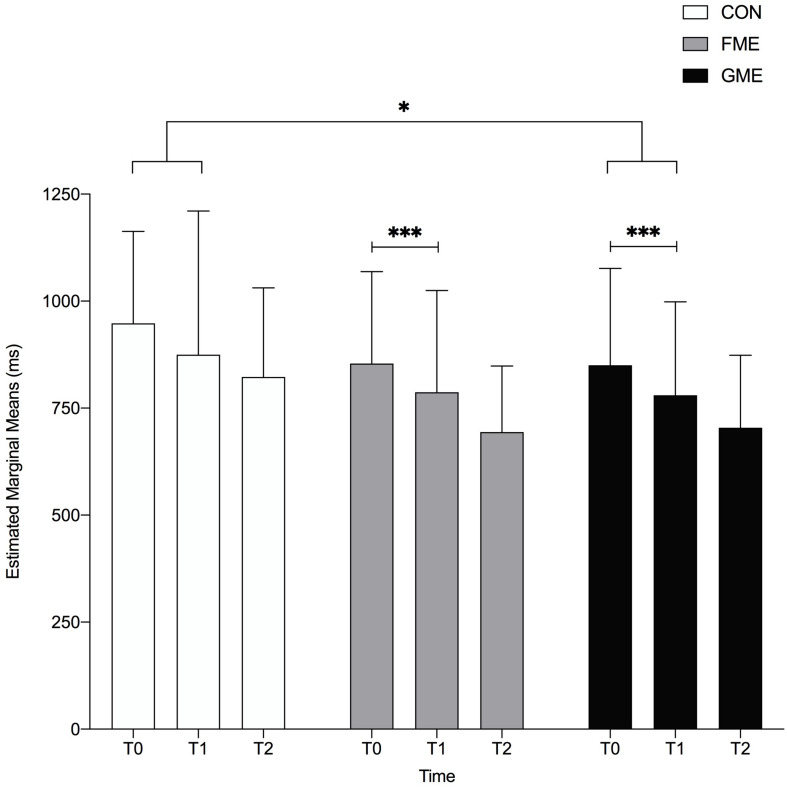
Reaction Time for Correctly Identified “b”/“d.” Data reported as estimated marginal means for reaction time (ms). CON, FME and GME improved their mean reaction time from T0 to T2 (*p* = 0.005, *p* < 0.001 and *p* < 0.001). GME and FME improved significantly from T0 to T1 (*p* = 0.001). The figure illustrates a significant better group-time improvement for GME compared to CON from T0 to T1 (*p* = 0.03). ^∗^, ^∗∗∗^ indicates significant improvement.

Likelihood Ratio Test did not reveal a global significant interaction between time and groups (*p* = 0.86) for reaction time of “b”/“d” recognition. However, planned comparisons revealed that GME and FME improved their mean reaction time significantly from T0 to T1 (*p* = 0.001), see Figure 4. A significant interaction was seen for GME compared to CON from T0 to T1 (*p* = 0.03). All three groups improved their mean reaction time significantly from T0 to T2 (CON; *p* = 0.007, FME; *p* < 0.001 and GME; *p* < 0.001).

Despite no global significant interaction between the time and the groups, there were an indication that motor-enriched learning (FME and GME) improved children’s mean reaction time for recognition of the letters “b” and “d” from T0 to T1 and from T1 to T2, and a larger improvement in reaction time for GME compared to CON from T0 to T1.

### Motivation

Significant differences between groups were found for the Intrinsic Motivation score (*p* = 0.003) using the Kruskall-Wallis rank sum test. Further, pairwise comparisons using Wilcoxon rank sum test showed differences between CON and FME (*p* < 0.01) and CON and GME (*p* = 0.01). Specifically, GME and FME had higher levels of intrinsic motivation for the activities compared to CON. No significant difference was observed between FME and GME (*p* > 0.1). No correlation was found between motivation score and accuracy in “b”/“d” Recognition Test (*p* = 0.79) and between reaction time in “b”/“d” Recognition Test and motivation score (*p* = 0.14).

## Discussion

### Effect of Fine Motor-Enriched Learning Activities on “b”/“d” Recognition

In this study, a superior positive retention effect of fine motor-enriched letter recognition training was found compared to non motor-enriched training. This result adds some support to the existing literature indicating that motor-enriched activities can enhance entrenchment of children’s academically relevant skills.

The improved effect of fine motor-enriched encoding might be a result of combinations of different mechanisms. It is proposed by Chandler and Tricot (2015) that movements integrated into a learning task can possibly influence children’s learning through various processes. It is known that attention to learning tasks is highly correlated with achievements, indicating that attention has a beneficial effect on outcomes, and can possibly be one of the reasons why the fine motor activities improve more than the other groups at T2 in letter recognition (Stewart et al., 2007). In this study, an immediate post intervention test (T1) and a one-day retention test (T2) were used to evaluate how well a specific skill was retained after a given time-interval. Based on knowledge of motor skill learning, the delayed retention is a better indicator of motor learning compared to performing right after end of practice (Kantak and Winstein, 2012; Roig et al., 2012). Our results confirmed similar improvements at retention evaluation as previously seen in motor skill learning. The intervention as well as the test are therefore related to conceptual knowledge.

### The Difference Between Fine Motor-Enriched Learning and Gross Motor-Enriched Learning in Letter Recognition

Our experiment indicated that a fine motor intervention had a greater positive effect on learning as indexed by correctness in “b”/“d” recognition than a gross motor intervention compared to non motor-enriched training. This is in contrast with previous studies (Beck et al., 2016; Pesce et al., 2016). A potential explanation for the discrepancy could be that the intervention in the present study (i.e., alternating right and left side motor responses) was more similar to the recall situation, which also had right and left side in the motor responses. Another explanation for the improvement in correctness for the fine motor intervention may be provided by the near hand theory. The near hand theory of activating bimodal cells through visual and tactile stimulation to provide spatial attention can explain why the FME group improved significantly more compared to CON from T0→T1 and T1→T2 which GME did not (Cléry et al., 2015; Bufacchi and Iannetti, 2018). The bimodal cells are of great importance for near hand space prioritization and can improve the attention and perception for visual stimuli near the hands. A study by Reed et al. (2006) found that both reaction time and accuracy were improved when stimuli occurred near the hands. In other words, using process selection for a near hand context in cognitive learning like the FME group may improve “b” and “d” recognition more than the non-near hand groups like gross motor movement and the control group (Wassenberg et al., 2005; Goodhew et al., 2015). This effect may help the children in the FME group to sustain attention during the intervention with a more impactful visual and tactile stimulus (Reed et al., 2006).

However, it is important to keep in mind that whereas the control group only used eye movements, the motor-enriched setup additionally involved motor control in relation to verbalization and limb movements. Therefore, we cannot exclude that verbalization also had an influence on the observed improved ability to recognize and distinguish “b”/“d” in the motor-enriched groups relative to the control group. If this would be the case, it should however influence performance in both motor-enriched groups, but the positive effect of motor enrichment was only observed for the FME group. This indicates that our main finding for the FME is not explained by the element of verbalization. It should be recognized as a limitation of the study, that the “b”/“d” Letter Recognition Test which was employed in the present study is a self-developed assessment tool, for which no validation procedure has been performed. Additionally, at one school data was not obtained for the T2 Letter Recognition Test due to practical issues. The generalized linear mixed model does however provide unbiased estimates.

### Reaction Time and Motor-Enriched Learning

We did not find any global significant interaction between time and the groups in children’s reaction time for letter recognition. However, the present study still found an indication that motor-enriched learning (FME and GME) improved children’s mean reaction time for recognition of the letters “b” and “d” from T0 to T1 and a better group-time improvement of reaction time for GME compared to CON from T0 to T1.

In the present study the intervention groups FME and GME had a larger coordinative activity than CON, which might have led to activation of the parts of the brain known to be responsible for mediating functions like attention (cerebellum and prefrontal cortex). Budde et al. (2008) found that 10-min bouts of whole-body coordinative exercise in a group of 13–16-year old enhanced attention and concentration (Budde et al., 2008). A recent study showed that coordinative physical activities improved children’s (8–11 years) attention measured with a d2-R test of attention (Gallotta et al., 2015). A study by Schmidt et al. (2016) investigated the effect of a 10-min cognitive challenging task compared to 10 min of physical activities showing that the cognitive challenging task group improved attention measured by a d2-R test, which neither the physical activity group did nor the group that combined the cognitive challenging task and physical activity did (Schmidt et al., 2016). The latter result of the study was unexcepted. The coordinative and challenging activity in especially our GME group may possibly have an influence on the immediate effect to attention leading to a possible explanation of a quicker mean reaction time seen in especially the GME. From the literature, we realize that perceptual movement manipulate the brain, mediate mental representations and memory (Madan and Singhal, 2012). A congruent mental motor stimulation is explained to accelerate the visual recognition (Helbig et al., 2006), which is in line with the present study seeing an improvement in reaction time for FME and GME with significant improvement from T0 to T1 and a significant change for GME compared to CON from T0 to T1.

### Intrinsic Motivation

Intrinsic motivation is impelled by many factors and is an important component to facilitate in teaching (Guay et al., 2010). A higher level of intrinsic motivation for the intervention activity was seen in motor-enriched groups compared to CON, indicating that motor-enriched teaching is more intrinsically motivating. No difference was seen between the FME and the GME. This finding is in agreement with Vazou and colleagues who found higher levels of enjoyment (a construct very similar to and closely related to intrinsic motivation) among pupils, after incorporating a 10-min single bout of acute moderate to vigorous physical activity in math lessons and also showed an improvement in reaction time at a standardized flanker task (Vazou and Smiley-Oyen, 2014). Our result on intrinsic motivation is also in line with an experimental study on having physical activity breaks when teaching different academic subjects which showed that integration of physical activity lessons were more intrinsically motivating to school children (Vazou et al., 2012). It is well established that intrinsically motivated behaviors are positively related to children’s psychological well-being, high-quality learning, academic achievement, and future academic success (Linnenbrink and Pintrich, 2002; Lepper et al., 2005; Burton et al., 2006). It is shown that intrinsic motivation is correlated with academic achievement, long-term performance, and wellbeing in children (Gottfried, 1990; Goldberg and Cornell, 1998; Broussard and Garrison, 2004). Therefore, the higher intrinsic motivation for the motor-enriched activities in the intervention groups FME and GME could have impacted both the acquisition of learning “b” and “d” in the intervention and the “b”/“d” letter testing session and thereby the outcome. However, several factors such as participant involvement, self-determination, the academic level, and relevance of the activity, all influence participants’ motivation (Deci and Ryan, 2002; Bugge et al., 2015).

## Conclusion

Children’s participation in 10 min of fine motor-enriched activities improved their ability to recognize and distinguish “b” and “d” compared to children who performed non motor-enriched activities. In general, improvement in letter recognition were seen in the motor-enriched activities compared to control. Both fine motor-enriched activities and gross motor-enriched activities resulted in a positive effect on children’s intrinsic motivation for performing the activities.

## Data Availability Statement

All datasets generated for this study are included in the article/Supplementary Material.

## Ethics Statement

The studies involving human participants were reviewed and approved by the Local Ethical Committee at UCPH. Written informed consent to participate in this study was provided by the participants’ legal guardian/next of kin.

## Author Contributions

LD, JW, AG, MP, AB, JL-J, and AM designed the experiment. LD, AG, JW, AB, and SE collected the data. LD and SE conducted the required data analysis. GN conducted the analysis for the motivation parameters. LD drafted the first version of the manuscript. All authors contributed to drafting the manuscript and approved the final version of the manuscript.

## Conflict of Interest

The authors declare that the research was conducted in the absence of any commercial or financial relationships that could be construed as a potential conflict of interest.

## Footnotes

1 https://CRAN.R-project.org/package=emmeans
